# Classifying Schizotypy Using an Audiovisual Emotion Perception Test and Scalp Electroencephalography

**DOI:** 10.3389/fnhum.2017.00450

**Published:** 2017-09-12

**Authors:** Ji Woon Jeong, Tariku W. Wendimagegn, Eunhee Chang, Yeseul Chun, Joon Hyuk Park, Hyoung Joong Kim, Hyun Taek Kim

**Affiliations:** ^1^Department of Psychology, Korea University Seoul, South Korea; ^2^Department of Information Security, Korea University Seoul, South Korea; ^3^Department of Neuropsychiatry, Jeju National University Hospital Jeju, South Korea

**Keywords:** Schizotypy, classification, EEG, multimodal emotion perception, shrinkage linear discriminant analysis

## Abstract

Schizotypy refers to the personality trait of experiencing “psychotic” symptoms and can be regarded as a predisposition of schizophrenia-spectrum psychopathology (Raine, 1991). Cumulative evidence has revealed that individuals with schizotypy, as well as schizophrenia patients, have emotional processing deficits. In the present study, we investigated multimodal emotion perception in schizotypy and implemented the machine learning technique to find out whether a schizotypy group (ST) is distinguishable from a control group (NC), using electroencephalogram (EEG) signals. Forty-five subjects (30 ST and 15 NC) were divided into two groups based on their scores on a Schizotypal Personality Questionnaire. All participants performed an audiovisual emotion perception test while EEG was recorded. After the preprocessing stage, the discriminatory features were extracted using a mean subsampling technique. For an accurate estimation of covariance matrices, the shrinkage linear discriminant algorithm was used. The classification attained over 98% accuracy and zero rate of false-positive results. This method may have important clinical implications in discriminating those among the general population who have a subtle risk for schizotypy, requiring intervention in advance.

## Introduction

Schizotypy is a pervasive pattern of intrapersonal and interpersonal deficits, characterized by perceptual, emotional, and cognitive distortions, as well as eccentric behaviors found on a schizophrenia spectrum (Chapman et al., 1994; Claridge et al., 1996; Kerns, 2006). The spectral view of psychosis, but not the categorical view, postulates that there is no clear-cut criterion between sanity and insanity (Eysenck, 1992; Claridge et al., 1996). Accordingly, schizotypy has been thought to have relatively mild positive and negative symptoms and dimensions, compared to those found in patients clinically diagnosed with schizophrenia (Gruzelier and Doig, 1996), and show different levels of cognitive and emotional impairments within certain contexts (Genetic Risk Outcome in Psychosis (GROUP) Investigators, 2011; Hori et al., 2012; Rossler et al., 2014). Therefore, studies of schizotypy in a normal population have provided a promising framework to understand the psychopathology of schizophrenia, as well as elucidating the psychobiological underpinnings of schizotypy itself, within the spectral view of psychosis.

One of the cardinal symptoms associated with schizophrenia concerns deficits in emotion perception. Patients with schizophrenia have consistently been reported to show deficits in recognizing emotions in facial (Borod et al., 1993; Hall et al., 2004; Kosmidis et al., 2007) and vocal (Bozikas et al., 2006; Leitman et al., 2010; Gold et al., 2012) expressions, and this finding is observed in both behavioral and electrophysiological studies (Campanella et al., 2006; Turetsky et al., 2007; Lynn and Salisbury, 2008; Wynn et al., 2008; Ramos-Loyo et al., 2009; Pinheiro et al., 2013).

For symptoms associated with schizotypy, recent neuroimaging studies have shown schizotypy have a mild level of emotional deficits. In an functional magnetic resonance imaging (fMRI) study, for instance, individuals with schizotypy showed emotion regulation difficulties, showing stronger activation in a number of prefrontal regions and decreased amygdala activation, when compared to controls, while reappraising emotion (Modinos et al., 2010). Further, the results of a study using emotional facial stimuli suggested the possibility that individuals with schizotypy have social interaction difficulties (Huang et al., 2013). Additionally, individuals with schizotypy have shown different patterns of brain activation to dynamically changed facial expressions compared to controls. This was especially true in the positive type of schizotypy, which showed deficits in emotion-cognition processes, showing qualitative alterations in neural processing patterns while performing an emotional Stroop task (Mohanty et al., 2005). Lastly, in an electroencephalogram (EEG) study, individuals with schizotypy showed impaired processing for social-emotional information. These individuals showed less prefrontal-posterior functional coupling than controls, which is considered to be indicative of a mechanism to protect the individual from becoming overwhelmed by the perception of social-emotion information, during exposure to auditory displays of strong emotion (Papousek et al., 2014).

As schizotypy is associated with a risk of developing schizophrenia, and some individuals with schizotypy become clinically ill, the discovery of a reliable method that can distinguish schizotypy from healthy individuals is vital. However, no reliable method has thus far been developed utilizing an objective measure for discriminating individuals with schizotypy from normal controls. Although, the Schizotypal Personality Questionnaire (SPQ; Raine, 1991) has been widely used for schizophrenia spectrum diagnoses, it is a self-report measure. Self-report measures are more often used for measuring levels of traits or symptom severity. Moreover, self-report questionnaires could suffer from social desirability bias, which can cause over-reporting of socially desirable and under-reporting of socially undesirable behaviors (Paulhus and Vazire, 2007).

In previous studies, EEG has been consistently used as a sensitive biomarker to distinguish individuals with impaired mental functioning, including Parkinson's Disease, Schizophrenia, and Alzheimer's Disease (AD), from controls (Kalatzis et al., 2004; Chapman et al., 2007; Lehmann et al., 2007; Sabeti et al., 2011; Yuvaraj and Murugappan, 2016). For instance, EEG was recorded in patients with Parkinson's disease with the left (LPD) or right side affected (RPD) and controls during the identification of six basic emotions: happiness, sadness, fear, anger, surprise, and disgust. Poorer classification performance using emotion-specific EEG features was shown in patients with LPD (inferred right-hemisphere pathology), which indicates that patients with LPD were more impaired in emotion processing compared to patients with RPD as well as controls. Moreover, EEG has been used to predict the progression to AD among individuals with Mild Cognitive Impairment (MCI), which is a transitional state between normal aging and AD (Chapman et al., 2011). In this study, a model was developed using an EEG signal for the discrimination of individuals with schizotypy and controls.

Several studies have shown the application of machine learning algorithms for the classifications of EEG signals (Cao et al., 2003; Wang and Paliwal, 2003; Liu et al., 2010; Subasi and Gursoy, 2010; Blankertz et al., 2011; Zhang et al., 2013; Chen et al., 2016). In this study, a supervised classification algorithm called the shrinkage linear discriminant analysis (SKLDA) was used to classify subjects based on their event-related potentials (ERP), since the sample size is much smaller than the dimensions of the feature vector. Previous research revealed that when the number of instances of the training data is much smaller than the dimensions of the feature vectors, a classifier could provide poor results (Jain and Chandrasekaran, 1982; Raudys and Jain, 1991). Researchers have recommended using, at least, 5–10 times as many training samples (per class) as the dimensionality. However, this is not easy for brain computer interface (BCI) applications because the dimensionality of the ERP feature vectors is usually much larger than the training set. It is possible to use other algorithms for dimensionality reduction, such as principal component analysis, to reduce the dimensionality significantly. However, EEG have a weak signal-to-noise ratio and their sensitivity to discriminatory features could easily be lost during the reduction. The SKLDA algorithm was proposed (Blankertz et al., 2011) to remedy this bias. The key advantage of SKLDA is to estimate an accurate covariance matrix that is particularly hard in studies with high dimensionality.

In the previous studies, a variety of tests using visual or auditory stimuli were used to investigate whether individuals with schizotypy have emotional deficits. However, as far as we know, there has been no investigation differentiating individuals with schizotypy from controls using a multimodal (audiovisual) emotion perception test. In a social context, emotional processing utilizing multiple sensory information is much more natural and common, as people rely on the visual (e.g., facial expressions and gestures) as well as the auditory modality (e.g., vocal tone, prosody, and accent) to judge the emotional states of others. Moreover, as individuals with schizotypy are considered to be non-clinical subjects, the heightened impact of emotionally laden inputs through a multimodal emotional test might produce significant differences between them and normal controls. Therefore, this study adopted a multimodal emotion perception test that might be theoretically appropriate as well as ecologically valid for assessing the brain function of individuals with schizotypy.

In summary, the objective of this study was to propose a reliable method to distinguish schizotypy from controls, based on measures of brain activity during emotional processing. To achieve this purpose, firstly, we developed a multimodal (audiovisual) emotion perception test, where subjects were asked to judge the emotions from both face and voice stimuli, presented simultaneously. We expected that performing a test with a higher emotional processing demand would allow for the assessment of brain functioning that may be affected by schizotypy. Furthermore, we adopted EEG methods, which can provide high temporal resolution. Whereas fMRI is temporally limited by the latency of the hemodynamic response, EEG directly measures the electric fields produced by neuronal activity (Dale et al., 2000). Therefore, it could be adequate for detecting brain activity during the emotional multisensory integration process. To identify individuals with schizotypy from controls based on their EEG data, we implemented the SKLDA. The details of the EEG data processing and features of extraction methods are presented in the sections below.

## Materials and methods

### Subjects

A set of questionnaires were administered online to 1,287 Korean university students. A set of questionnaires included a consent form, demographic questions, the SPQ, and the Center for Epidemiological Studies Depression Scale (CES-D; Radloff, 1977).

The SPQ is a 74-item scale (score ranges: 0–74) modeled on DSM-III-R criteria for schizotypal personality disorder. One hundred and five subjects who scored in the top 10% on the SPQ (scores of ≥ 31) were selected as the schizotypy (ST) group, and 464 subjects who scored within ±0.5 standard deviation (*SD*) from the mean (*M*) score on the SPQ were selected as the normal control (NC) group. Among them, 51 subjects from the ST group and 45 subjects from NC group were excluded based on scores of the CES-D (i.e., scores ≥ 25) because depression was reported to be comorbid with schizophrenia (Lewandowski et al., 2006) and could influence on facial emotion recognition (Feinberg et al., 1986; Persad and Polivy, 1993).

Thirty-four subjects from the ST group (age: *M* = 20.83, *SD* = 2.42; % female: 57.22) and 17 subjects from NC group (age: *M* = 21.06, *SD* = 1.60; % female: 50.00) were randomly selected to participate in the experiment among the 513 subjects (ST: 94, NC: 419). Among them, data from 6 subjects (4 NC and 2 ST) were excluded from the analyses because of excessive electrical artifacts during EEG recordings. Mean SPQ scores from ST and NC groups were 37.35 (*SD* = 7.57) and 15.53 (*SD* = 3.12), respectively. All subjects gave written informed consent in accordance with the Declaration of Helsinki. The protocol was approved by the Ethics Committee of Korea University (1040548-KU-IRB-14-75-A-3). All subjects had normal or corrected to normal vision and no history of neurological and/or psychiatric disorders.

### Audiovisual emotion perception test (AEPT)

**Figure 1:**
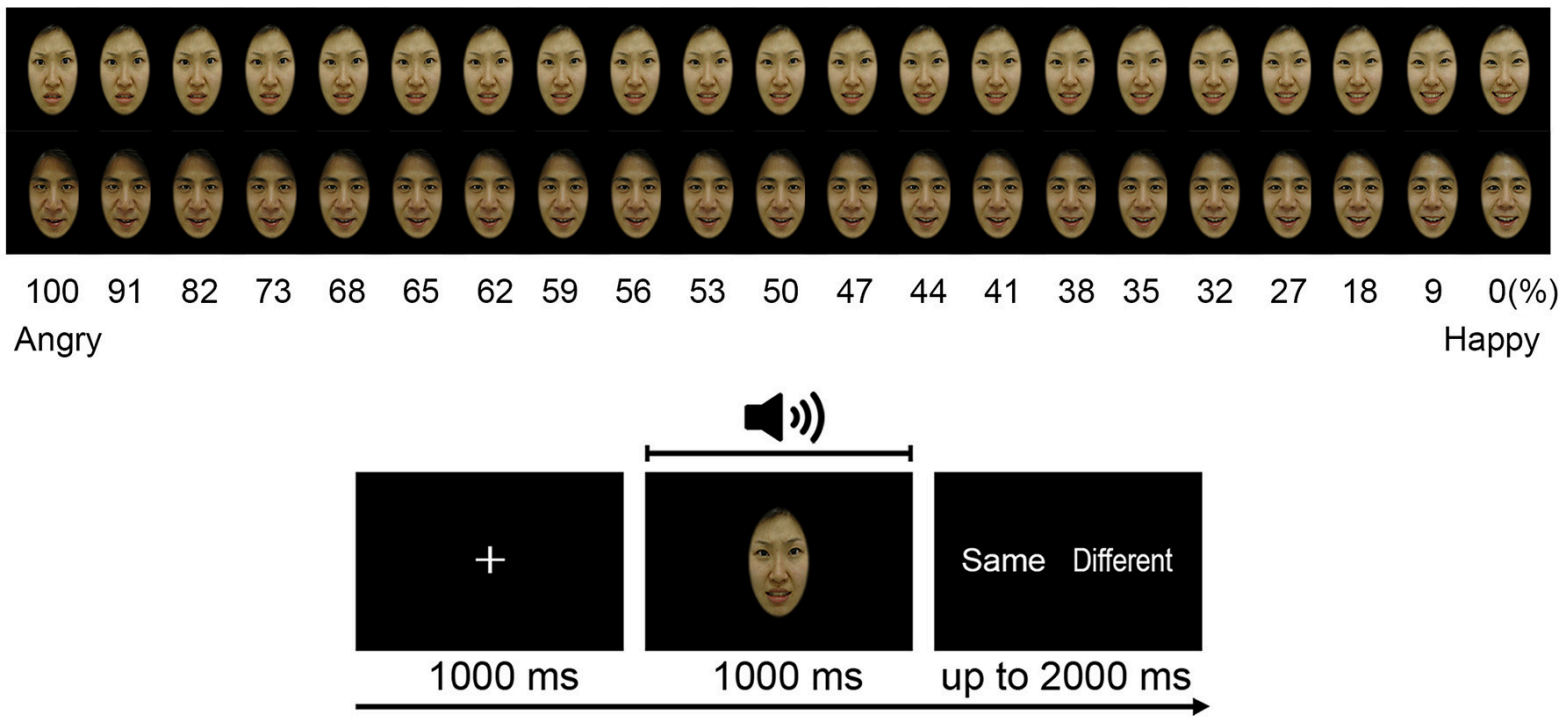
Audiovisual emotion perception test. Subjects were asked to judge whether the emotions on the face and the voice were same or not. Figure shows morphed face images used at the AEPT **(upper)** and the AEPT trial sequence **(lower)**.

Figure 1 illustrates the Audiovisual emotion perception test. The facial images (2 males, 2 females) were created from the Korea University Facial Expression Collection (KUFEC; Kim et al., 2011). Photoshop CS5 (Adobe systems, USA) was used to crop the photographs, remove non-facial attributes (e.g., hair, ears), and create a uniform black background. A computerized morphing program (Abrosoft FantaMorph version 5.4.1; Abrosoft, USA) was used to create a linear continuum of facial images. Morphed faces were created by merging two face pictures (e.g., angry face and happy face) in 1% steps, resulting in 101 morphed face images (e.g., from 0 to 100% happy), with the graded blending of the facial features of the two faces. Two prototypical images (e.g., 100% angry and happy faces), 6 unambiguous images near to the prototypical images (91, 82, 73, 27, 18, and 9% morphed faces), and 13 ambiguous images in the midrange (68, 65, 62, 59, 56, 53, 50, 47, 44, 41, 38, 35, and 32% morphed faces) were selected from the continuum and used for the AEPT.

Vocal stimuli of happy and angry emotional valence were recorded by four amateur actors (two males, two female) in a noise-free room. The actors were instructed to pronounce four semantically neutral sentences either in an angry or happy voice since content cues could help in identifying the emotion. Four Korean sentences were “stayed in the house,” “went by airplane,” “will be going to Seoul,” and “arrived in Busan.” The mean length of the 16 voice stimuli (4 sentences × 2 actors × 2 emotions) was 1.06s (*SD* = 0.059).

The AEPT consisted of 840 trials (4 identities × 21 morphed images × 2 voice emotions × 5 repetitions), which were 400 congruent (e.g., angry face/angry voice or happy face/happy voice audiovisual stimulus pairs), 400 incongruent (e.g., angry face/happy voice or happy face/angry voice audiovisual stimulus pairs), and 40 trials with no correct answer (e.g., 50% morphed face/happy voice or 50% morphed face/angry voice audiovisual stimulus pairs). These 40 trials were excluded from the data analysis. In the test, subjects were asked to judge whether the emotions on the face and the voice were same (congruent) or not (incongruent).

**Figure 2:**
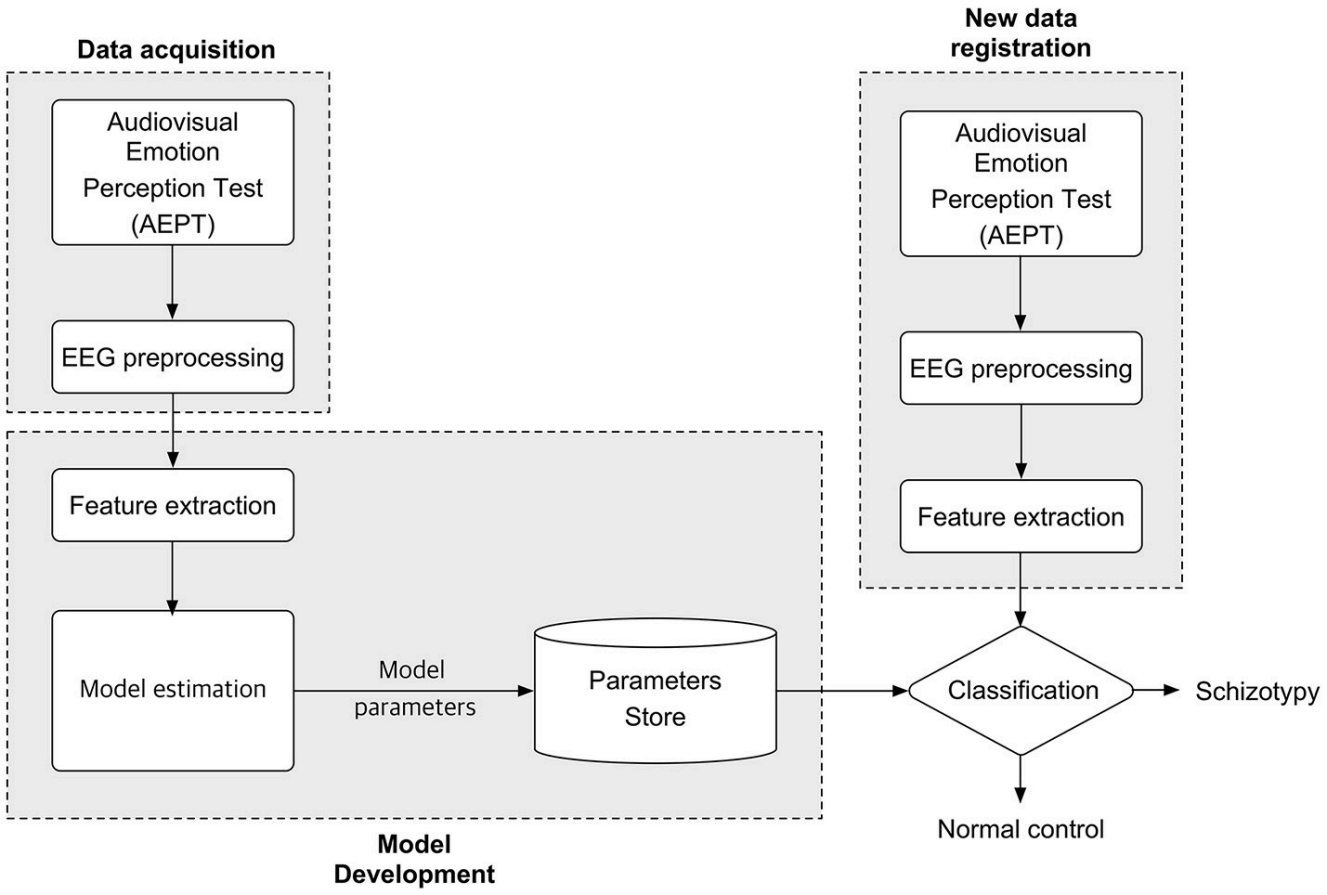
Flowchart for the proposed classification approach.

A trial started with the presentation of a 1,000 ms fixation, which was then followed by the presentation of the audiovisual stimulus pairs. The face was presented for 1,000 ms, while the voice was delivered via earphone. When the length of the voice was longer than 1,000 ms, a black blank screen was briefly presented on the screen for the rest of the duration. Subjects pressed either a “same” or “different” button during the subsequent 2,000 ms. Subjects pressed buttons using one hand, and response hands were counterbalanced across subjects. The flowchart for the proposed classification approach is depicted in Figure 2.

### EEG recordings and preprocessing

EEG was recorded continuously from 14 electrodes (Fz, Cz, Pz, OZ, F3, F4, C3, C4, P3, P4, O1, O2, T5, and T6) using a Grass Model 12 Neurodata Acquisition System (Grass Technologies Astro-Med, Inc., West Warwick, USA) according to the extended 10–20 system. The vertical electrooculogram (EOG), which records the voltage difference between two electrodes placed above and below the left eye, was used to detect eye blinks. A single ground electrode was placed on the forehead, and the reference electrode was located at the right earlobe. The signals were recorded continuously at a sampling rate of 1,024 Hz (bandpass filter, 0.01–100 Hz). The electrode impedances were below 5 kΩ.

The offline data analysis was performed with EEGLAB (version 12.0.2.2) running under Matlab 2012a (Mathworks, USA; MATLAB, 2012). All EEG data were re-referenced to linked earlobes. Gross movement artifacts were removed from the data based on visual inspection. The data were digitally filtered with a band pass of 0.1–30 Hz. The data were epoched between −200 and 800 ms, relative to stimulus onset. The epochs were baseline-corrected, and those containing artifacts larger than ±50 μV were removed. Individual EOG artifact correction was conducted using an independent component analysis. Data was then down sampled to 350 data points by taking mean of four consecutive data points.

### Behavioral and event-related potentials analysis

The epochs were separately averaged for the ST and NC groups to obtain ERP. The assumptions for the normality and homogeneity of variance were tested with the Shapiro–Wilk and Levene's test, respectively, for both behavioral and ERP data. For the behavioral data analysis, one-way ANOVAs were performed on the percent of correct responses and reaction times. For the ERP data analysis, mixed-model analysis of variance (ANOVA) for averaged ERP amplitude in a time window ranging from 150 to 270 ms (P2 component) was performed, in which site (14) was the within-subjects factor and group (2) was the between-subjects factor. The Greenhouse-Geisser adjustment was used to correct for violations of sphericity.

### Feature extraction

Let the ERP at channel c that varies as a function of time ***t*** be denoted by *X*_*c*_(*t*). Assume that a trial lasts for T time units, where ***T*** = {*t*_1_, *t*_2_, *t*_3_, …, *t*_*T*_}, and is sampled at equally spaced intervals. Let *C*_*i*_(t) be the potential of the *i*^*th*^ channel at time point ***t***. Then, the vector *X*_*C*_*i*__(***T***) = [*c*(*t*_1_), *c*(*t*_2_), …, *c*(*t*_*N*_)] defines the ERP of this channel for the duration **T**. Given *C* = {*C*_1_, *C*_2_, …, *C*_*M*_}, as a subset of channels chosen for analysis, where for *C*_*j*_, *j* = 1, 2, 3, …, and *M* represents a chosen channel. Equation (1) defines a vector of potential values for the subset of channels at time point ***t*** for the *k*^*th*^ trial, and **τ** denotes vector transpose.

(1) $${X_{C}}^{K}\left(t\right)={\left[{X_{C_{1}}}^{K}\left(t\right),\;{X_{C_{2}}}^{K}\left(t\right),{X_{C_{3}}}^{K}\left(t\right)\ldots ,{X_{C_{M}}}^{K}\left(t\right)\right]}^{\tau }$$

Concatenating those vectors for all time points of ***T*** across all chosen channels, for the *k*^*th*^ trial gives the spatio-temporal features shown in Equation (2).

(2) $${X_{C}}^{K}(T)=[{X_{C_{1}}}^{K}\left(T\right),\;{X_{C_{2}}}^{K}\left(T\right),{X_{C_{3}}}^{K}\left(T\right)\ldots ,{X_{C_{M}}}^{K}\left(T\right)]$$

For the sake of readability, notations are simplified as follows: both *C* and *T* (constants) are omitted, and ${X_{C}}^{K}\left(T\right)$ is denoted by *X*^*K*^. Hence, *X*^*K*^ ϵ *R*^*M* × *N*^ represents spatiotemporal data of one participant for the *k*^*th*^ trial. The data set is grouped into three sets based on the stimuli used in the AEPT test. The first set contains all trials for happy visual stimuli only. Similarly, the second set contains all data from angry stimuli only, and the third set contains all data from both happy and angry stimuli trials. Let *x*^*H*^, *x*^*A*^, *and x*^*HA*^ denote the data set of happy only, angry only, and both happy and angry stimuli, respectively. Then, define the averages of the *i*^*th*^ participant's data over stimuli type using Equation (3).

(3) $$\begin{array}{l} {X_{i}}^{h}=\frac{1}{N_{H}}\sum_{{x_{i}}^{k}\in x^{H}}{x_{i}}^{k}\\ {X_{i}}^{a}=\frac{1}{N_{A}}\sum_{{x_{i}}^{k}\in x^{A}}{x_{i}}^{k}\\ {X_{i}}^{ha}=\frac{1}{N_{HA}}\sum_{{x_{i}}^{k}\in x^{HA}}{x_{i}}^{k} \end{array}$$

Here, ${x_{n}}^{m}$, *m* ϵ*{a, h, ha}* defines the mean of the *i*^*th*^ participant samples over stimuli type; *N*_*H*_ and *N*_*A*_ is the number of trials with happy and angry stimuli, respectively, and *N*_*HA*_ = *N*_*H*_ + *N*_*A*_. As explained earlier in this section, ***T*** has equally spaced intervals. Hence, dimensionality reduction achieved by mean subsampling (replacing the ***n*** consecutive data points by their mean), where ***n*** is a heuristically determined constant.

To be specific to this study, ERP values from 0 ms (right after onset of stimuli) to 800 ms after stimuli onset were chosen for analysis. The sampling rate was reduced from 1,024 to 256 Hz using mean subsampling (for *n* = 4). After down sampling, (8001000×1024)4=204 features were extracted per channel. For the analysis, the first 200 features were selected. In total, 2,800 features were extracted from the 14 channels and utilized as input during classification.

### Classification

#### SKLDA

The conventional LDA has been widely adopted for feature reduction during the classification of ERP for BCI applications (Lehmann et al., 2007; Lotte et al., 2007; Subasi and Gursoy, 2010). Due to the high similarity of covariance matrices in the Gaussian distribution corresponding to the features of ERP and non-ERP (i.e., targets and non-targets), LDA performs well for the classification of ERP (Blankertz et al., 2011) and it can be described using the Rayleigh Equation, as defined by Equation (4).

(4) $$J\left(W\right)=\frac{W^{T}S_{b}W}{W^{T}S_{w}W}$$

where *S*_*b*_ and *S*_*w*_ are the between and within class scatter matrices, respectively; *W* is the projection vector and *T* is the transpose. The optimum transformation is obtained by the maximization of $W^{T}S_{b}W$ and the minimization of $W^{T}S_{w}W$.

The empirically estimated covariance is a standard estimator for the covariance matrix. This estimator is unbiased and has good properties under usual conditions. However, it may give an imprecise estimation if data with high dimensionality and low sample size are used for training. This is because the number of unknown parameters that have to be estimated is quadratic in the number of dimensions, leading to a systematic error. The systematic error causes estimation of large eigenvalues of the covariance matrix to be very large and estimation of small eigenvalues to be very small.

Shrinkage of the estimated covariance matrix is a way of correcting this systematic bias. The SKLDA can reduce the ill-conditioned covariance matrix with an appropriately selected shrinkage parameter, and can effectively enhance the generalization capability of the classifier, thereby providing more accurate classification of ERP, even when using insufficient training samples (Blankertz et al., 2011). The SKLDA is an accurate covariance matrix estimator, particularly useful in high dimension studies. Let $X_{\text{1}},X_{\text{2}},X_{\text{3}},\ldots ,X_{\text{n}}\in R^{d}$ be ***n*** feature vectors with dimension *d*. Denote the unbiased estimator of the covariance matrix as $\tilde{\sum }$. Then, a shrinkage parameter gamma, γ is used to relate $\hat{\sum }$ to $\tilde{\sum }$ as defined in Equation (5).

(5) $$\tilde{\sum }(\gamma )=\left(1-\gamma \right)\hat{\sum }+\gamma vI$$

In this equation, γ ϵ [0, 1] is the shrinkage parameter, $\tilde{\sum }$ and $\hat{\sum }$ are the shrinkage and empirical covariances, respectively, and ν is the average eigenvalue of the empirical covariance matrix, $v=trace\left(\hat{\sum }\right)/d$; *d* denotes the dimensionality of the data, and *I* the identity matrix. The fact that $\;\tilde{\sum }=\hat{\sum }$, when γ = 0 means that the sample-based estimated covariance can accurately measure the variability of the training sets, and no shrinkage is required. However, $\tilde{\sum \;}$ = γ*vI* when γ = 1, means the estimated covariance matrix $\tilde{\sum }$ poorly measures the variability in the sample. Heuristically, γ is set as γ*ϵS* = {0, 0.05, 0.2, 0.4, 0.6, 0.8, 1.0}, considering its linear property and using a heuristic approach. Schäfer and Strimmer (2005) provide the analytical solution of γ for reference. In this paper, nested-cross-validation is used to estimate γ.

#### LOOCV

The leave-one-out cross-validation (LOOCV), also known as nested-cross-validation, is used for classifier evaluation. LOOCV works as follows: (1) group the entire training set into *N*-folds; (2) holding first-fold out, again group the data from the remaining N−1 folds into M-folds; (3) holding the first fold from the M-folds out, train a model (classifier) using the data from the M-1 folds; (4) score (predict) the first-fold from the second step using the developed model. Keep this classifier and its accuracy; (5) repeat this from the third step onward for every fold; (6) choose the model giving the minimum classification error rate from the models in fourth step as a candidate classifier; (7) score (validate) the first-fold in step one using the candidate classifier obtained in step six. Repeat from step one for the remaining folds. The steps produce *N* scores that do not capitalize on one chance. Choose the model with the highest classification accuracy. The assumption is that classifiers showing higher classification accuracy on the testing data are more likely to have higher classification accuracy on the validation data.

**Figure 3:**
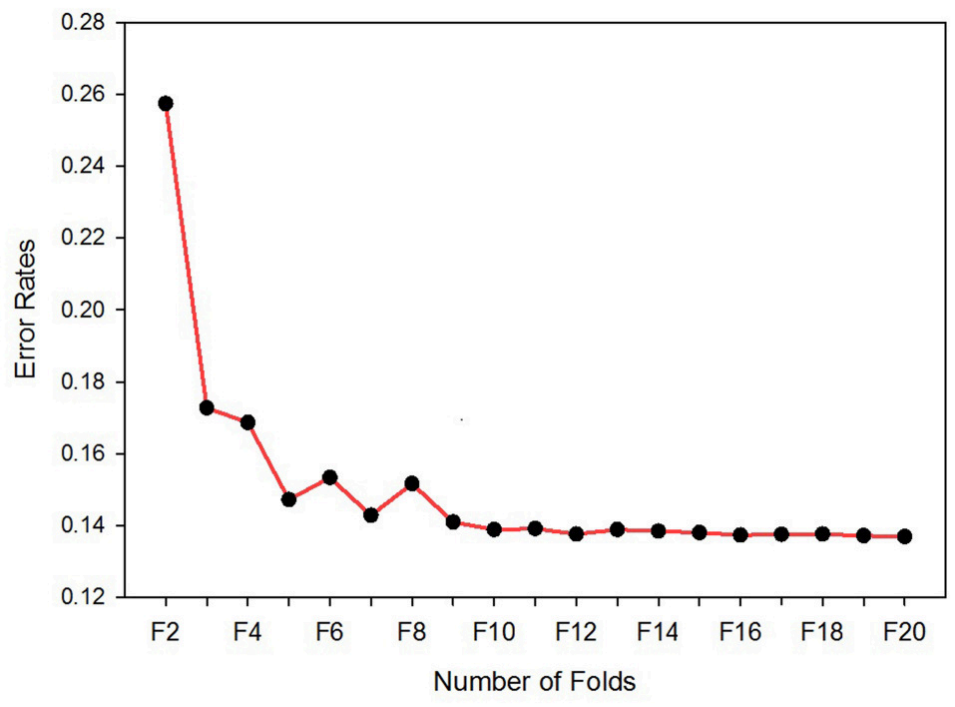
Error rates vs. number of folds. The optimum number of folds for the cross-validation determined by running the classifier for 20 times per fold.

The number of folds used for the cross-validation is decided on by running the classifier for 20-folds, starting from 2- to 20-folds. Figure 3 depicts the error rates versus number of folds. The experimental result shows that as the number of folds increased, the error rate decreased rapidly, up until the ninth-fold. However, from the tenth-fold onwards the error rate decreased very slowly. Taking into consideration the iteration cost and the error rate, the tenth-folds is used for cross-validation. Stratification is used to split the sample sets.

#### Performance measures

To evaluate the correctness of a classifier, some statistical performance measures can be used. These measures depend on the four entries of a confusion matrix, namely, the number of correctly recognized class examples (true positives, TP), the number of correctly recognized examples that do not belong to the class (true negatives, TN), and examples that either were incorrectly assigned to the class (false positives, FP) or those that were not recognized as class examples (false negatives, FN). Based on the four terms, the following performance measures were used to evaluate the classifier.

Accuracy: the fraction of correct classifications. It summarizes the overall effectiveness of the classifier.

(6) $$Accuracy=\frac{\rho TP+TN}{\rho TP+TN+FP+\rho FN}\times 100\%$$

Recall: proportion of actual positives, which are predicted positive. Also known as sensitivity.

(7) $$Recall=\frac{TP}{TP+FN}\times 100\%$$

Precision: proportion of predicted positives which are actual positive.

(8) $$Precision=\frac{TP}{TP+FP}\times 100\%$$

Specificity: fraction of those negatives that will have a negative test result. Also referred to as true negative rate.

(9) $$Specificity=\frac{TN}{TN+FP}\times 100\%$$

F1-measure (weighted harmonic mean): combined measure that assesses precision/recall tradeoff. It provides a relation between the positive labels of the data and those given by a classifier.

(10) $$F1\;measure=\frac{2}{1/precision+1/recall}\times 100\%$$

where ρ is an adaptive normalization constant used for imbalanced data. It is computed as the ratio of the number of samples in the smaller sample size to the number of samples in the larger sized class.

#### Channel selection

To choose the smallest subset of the 14 channels with the highest discriminatory information, a systematic selection algorithm is required. For this, the sequential forward selection (SFS) greedy algorithm (Marcano-Cedeño et al., 2010; Liu et al., 2015) can be used. The SFS algorithm starts with an empty list. Pairs of channels giving the highest classification accuracy are searched for and added to the empty list. Next, a third channel satisfying two conditions is obtained. There are two important aspects to this; first, this channel should give the highest classification accuracy when combined with the previously chosen channels; second, the classification accuracy of the three channels must be greater than the classification accuracy of the previously chosen channels. Repeat the steps until adding any more channel tends to decrease the performance.

## Results

### Behavior

Finally, 30 ST and 15 NC were included in the data analyses. The percentage of correct responses (%) was not significantly different between the ST (*M* = 81.73, *SD* = 5.17) and NC (*M* = 83.97, *SD* = 4.23) groups [*F*_(1, 44)_ = 0.050, *p* = 0.83]. The reaction time (ms) was also not significantly different between the two groups [*M* = 414, *SD* = 138 for ST; *M* = 424, *SD* = 135 for NC; *F*_(1, 44)_ = 2.097, *p* = 0.16].

### ERP

**Figure 4:**
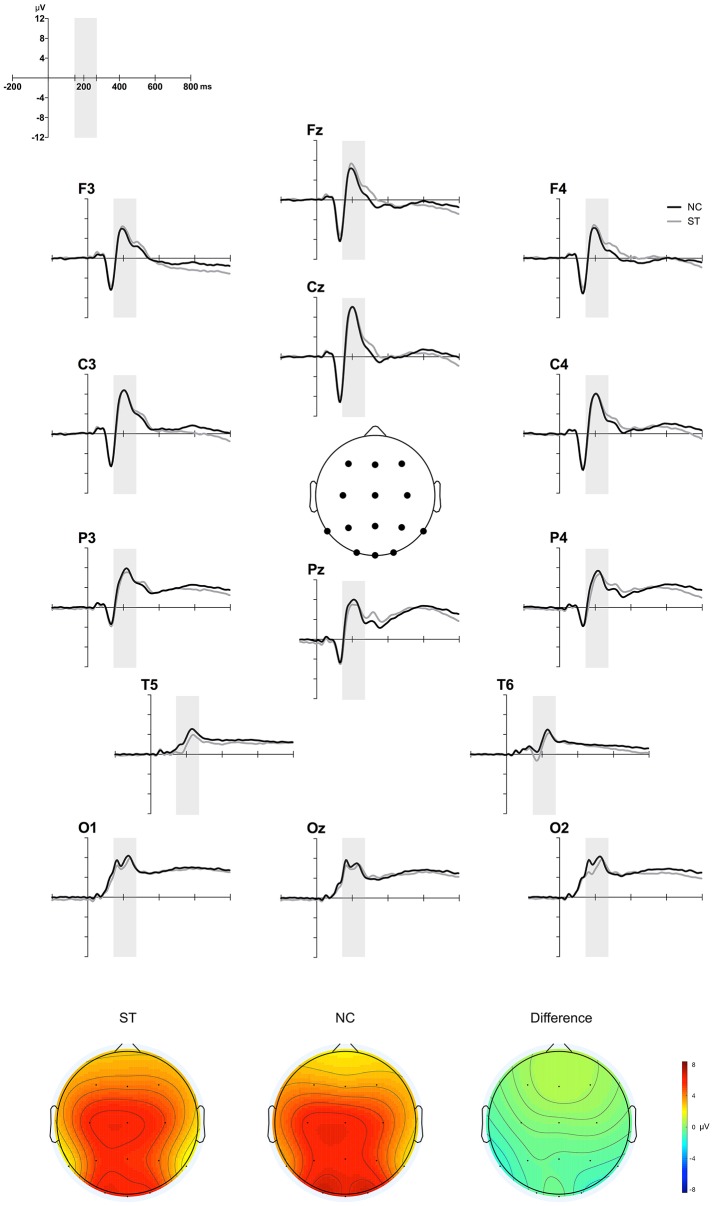
ERP results. Figure shows the grand-averaged ERP waveforms during the AEPT for the ST and NC at 12 sites **(upper)** and the NC minus ST difference topographical maps in the 150–270 ms time window **(lower)**.

Figure 4 depicts the grand-averaged ERP waveforms at all electrode sites (upper) and the NC minus ST difference in topographies in the 150–270 ms time window (lower). A group × site ANOVA showed a significant site effect [*M* = 3.88, SE = 0.49 for Fz; *M* = 3.89, SE = 0.42 for F3; *M* = 3.77, SE = 0.49 for F4; *M* = 6.23, SE = 0.55 for Cz; *M* = 5.96, SE = 0.42 for C3; *M* = 5.11, SE = 0.47 for C4; *M* = 5.73, SE = 0.51 for Pz; *M* = 5.55, SE = 0.36 for P3; *M* = 4.86, SE = 0.42 for P4; *M* = 6.25, SE = 0.47 for Oz; *M* = 6.88, SE = 0.51 for O1; *M* = 6.57, SE = 0.45 for O2; *M* = 2.89, SE = 0.26 for T5; *M* = 2.32, SE = 0.29 for T6; *F*_(13, 559)_ = 20.69, *p* < 0.001], but not a significant group effect [*M* = 4.82, SE = 0.38 for ST; *M* = 5.16, SE = 0.53 for NC; *F*_(1, 43)_ = 0.27, *p* = 0.60]. Interaction effects were not observed [*F*_(13, 559)_ = 1.01, *p* = 0.39]. These results suggest that P2 component, an indicative of multisensory processing, was not different between two groups.

### Classification

**Figure 5:**
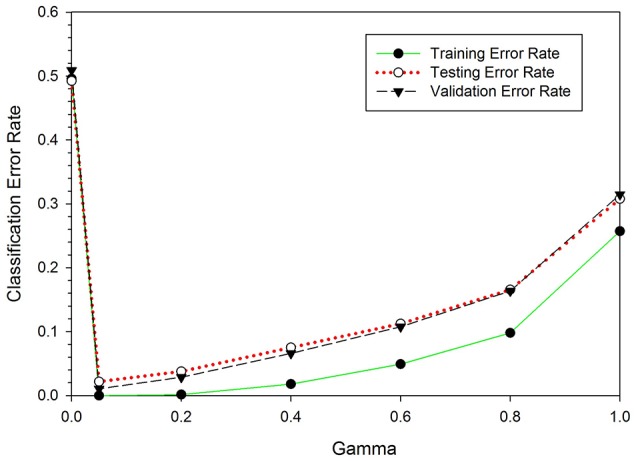
Classification error rates as a function of the shrinkage parameter, γ. When γ increased from 0 to 0.05, the classification error rates dropped from 0.55 to 0.04. However, as γ increased from 0.05 to 1.0 the classification error rates increased from 0.04 to 0.31. For all three curves, the error rate is minimum at γ = 0.05.

The SKLDA algorithm was implemented through the Matlab2012a working environment. The classifier was trained using EEG data from the NC and ST groups; it was evaluated using LOOCV and the performance measures provided in Section LOOCV. As mentioned earlier, one key advantage of SKLDA is an accurate estimation of covariance matrices; this is particularly useful for high dimensional data. Before estimating the covariance, the shrinkage parameter value was computed. Figure 5 shows the classification error rates as a function of the shrinkage parameter gamma, γ. In this figure, the vertical and the horizontal axes represent the classification error rates and the shrinkage parameters, respectively. The three curves represent the misclassification rates for the training (green), testing (red) and validation (blue) sets. All three curves attain their minimum at γ = 0.05, verifying the robustness for the value of γ. Considering the convexity of Equation (4), and principles of calculus for first derivatives, the optimal value of γ was estimated to be 0.05. Table 1 presents performance rates of the classifier for the validation set as a function of γ. When the value of γ increased from 0.00 to 0.05, the classification accuracy increased from 50% to over 98%. Similarly, the values of the precision (Pr), recall (Re), specificity (Sp), and F1-measure (F1) also increased. However, when γ increased from 0.05 to 1.0, the classification accuracy and other performance measures decreased. Therefore, we chose γ = 0.05 as an optimal value for the shrinkage parameter. Unless mentioned otherwise, all reports in this document are based on the value of γ = 0.05.

**Table 1:** Performance rates (%) of the classifier for the validation.

**Gamma**	**Pr**	**Re**	**Sp**	**F1**	**Ac**
0.00	51.47	50.17	50.84	50.30	50.52
0.05	98.18	99.21	97.64	98.41	98.61
0.20	98.14	98.88	97.59	98.34	98.17
0.40	98.31	98.77	97.83	98.38	98.01
0.60	98.17	98.06	97.71	97.94	97.09
0.80	96.09	95.26	95.22	95.29	93.90
1.00	66.16	72.12	59.51	67.92	68.64

**Figure 6:**
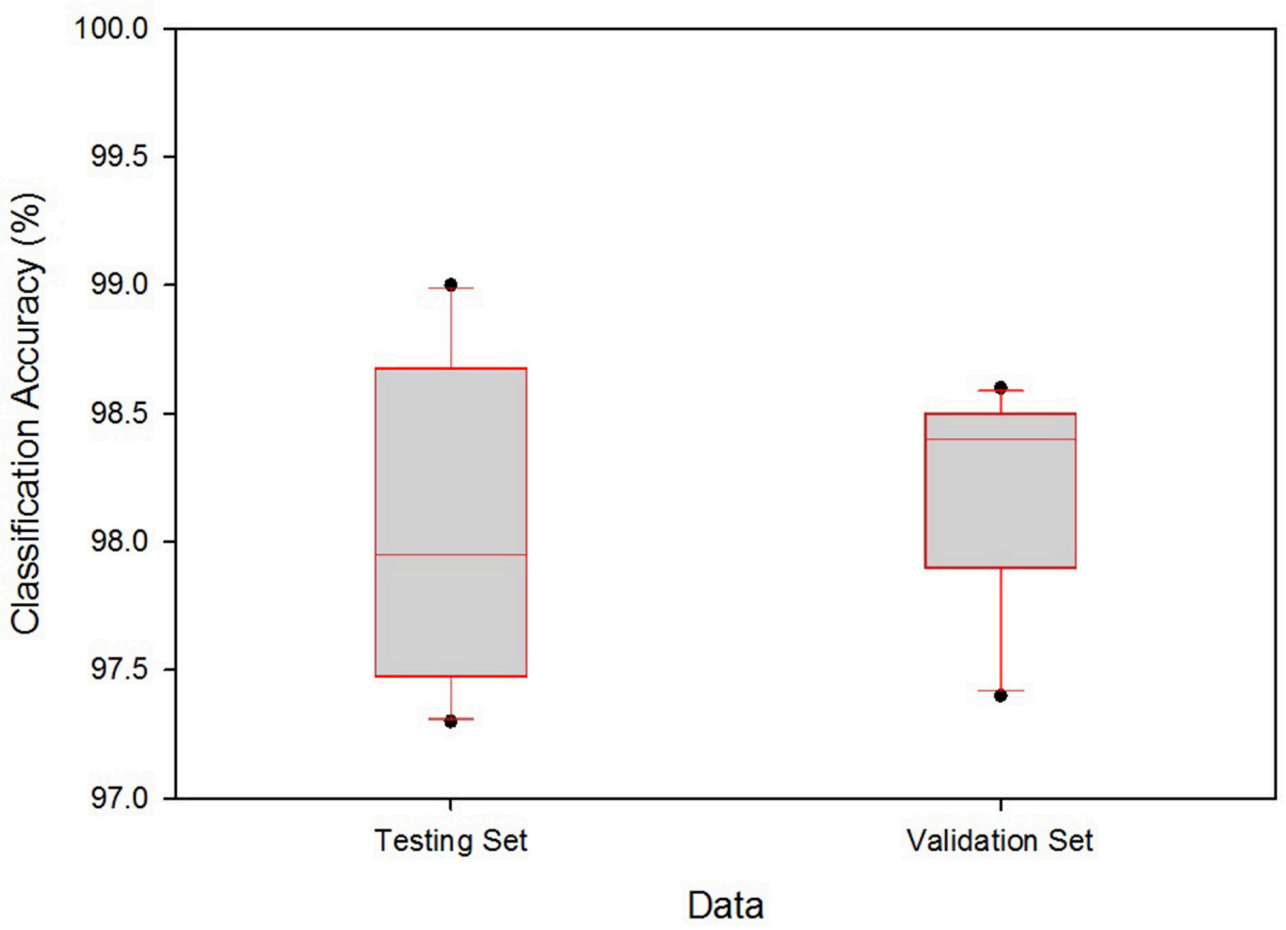
Boxplot for the range of classification accuracy rates at γ = 0.05.

The boxplot in Figure 6 shows the range of classification accuracy rates at γ = 0.05. The vertical axis denotes the range of classification accuracy rates for the testing and validation data. From the given boxplots, the testing set accuracies range from 0.9732 to 0.99, i.e., for the testing set, the minimum accuracy is 0.9732 ≅ 97.32% and the maximum is 99.0%. Similarly, the minimum and the maximum classification accuracy for the validation set are 97.42 and 98.56%, respectively. From this figure, one can see that the accuracy range for the cross-validation is smaller than that of the testing accuracy range which supports the assumption given in Section LOOCV, i.e., best classifiers for the testing can do better for the validation set.

Table 2 presents the classification accuracies for both the training (TR) and testing (TS) sets. Channels found at the top of this table have the highest classification accuracies (smallest error rate) and those found near the bottom of this table have the least classification accuracies. Similar to the case of using 14 channels at once, these classifiers acquired their minimum classification error at γ = 0.05.

**Table 2:** The classification accuracies for both the training (TR) and testing (TS) sets.

**Gamma/channel**	**0**		**0.05**		**0.2**		**0.4**		**0.6**		**0.8**		**1.0**	
	**TR**	**TS**	**TR**	**TS**	**TR**	**TS**	**TR**	**TS**	**TR**	**TS**	**TR**	**TS**	**TR**	**TS**
P4	0.5079	0.5032	0.9236	0.8435	0.8956	0.8419	0.8741	0.8225	0.8523	0.7988	0.8183	0.7622	0.7007	0.6667
Pz	0.4979	0.4845	0.9161	0.835	0.8808	0.8332	0.8384	0.783	0.7787	0.7429	0.7200	0.7151	0.6494	0.6385
T5	0.4686	0.5138	0.9167	0.8345	0.8775	0.8061	0.8388	0.7709	0.8025	0.7262	0.7695	0.702	0.6334	0.5828
T6	0.5143	0.4686	0.9026	0.8223	0.8598	0.8023	0.8423	0.8062	0.8248	0.7893	0.8029	0.7744	0.7365	0.6954
O2	0.4879	0.5033	0.9217	0.8156	0.8871	0.7977	0.8543	0.7791	0.817	0.7565	0.7834	0.7312	0.7461	0.738
Oz	0.5014	0.4792	0.9102	0.7912	0.8637	0.7666	0.8211	0.7196	0.7748	0.695	0.737	0.6909	0.6448	0.5741
P3	0.5014	0.5044	0.9157	0.7809	0.8616	0.7194	0.8033	0.6507	0.7544	0.6122	0.7143	0.5826	0.6298	0.5501
O1	0.4964	0.5008	0.8959	0.7780	0.8347	0.7513	0.7892	0.7086	0.7474	0.6651	0.7007	0.6053	0.6038	0.5200
Cz	0.5207	0.4899	0.8561	0.7560	0.7997	0.712	0.7476	0.6642	0.7196	0.6382	0.6949	0.6232	0.6390	0.5764
C3	0.5143	0.5045	0.8522	0.7380	0.8113	0.6804	0.7664	0.6248	0.7295	0.5939	0.6902	0.5771	0.5690	0.5819
F4	0.4857	0.5041	0.8594	0.7271	0.7992	0.6673	0.7556	0.627	0.7261	0.5882	0.6844	0.5586	0.5408	0.4833
Fz	0.5251	0.4676	0.8389	0.7208	0.7673	0.6639	0.7288	0.6326	0.6999	0.6140	0.6717	0.5835	0.6096	0.5246
C4	0.4757	0.492	0.8462	0.7158	0.7836	0.6806	0.758	0.6639	0.7355	0.6470	0.7111	0.6168	0.6624	0.5845
F3	0.4911	0.5221	0.8333	0.6552	0.7735	0.5918	0.7198	0.5532	0.6761	0.5356	0.6336	0.5161	0.5659	0.5525

Using the SFS greedy selection algorithm, we found that three channels (P4, Pz, and T5) perform very similarly to the 14 channels, but with minimum computational cost for the developmental phase. Using the selected channels, we obtained a classification accuracy of 98.18%, which is approximately equal to the accuracy obtained while using all the 14 channels (98.22%) at once. Table 3 presents the results of SFS algorithm.

**Table 3:** Performance rate (%) of channel combination.

**Channel**	**Pr**.		**Re**		**Sp**.		**F1**		**Acc**.		**No. of chan**.
	**TR**	**TS**	**TR**	**TS**	**TR**	**TS**	**TR**	**TS**	**TR**	**TS**	
P4	0.9875	0.9080	0.8996	0.8390	0.9882	0.8972	0.9412	0.8637	0.9224	0.8446	1
P4/Pz	0.9997	0.9555	0.9744	0.9099	0.9997	0.9479	0.9868	0.9266	0.9811	0.9078	2
All Except F3	1.0000	0.9749	1.0000	0.9917	1.0000	0.9666	1.0000	0.9813	1.0000	0.9806	13
P4/Pz/T5/T6/ O2/Oz/O1/P3 /C3	1.0000	0.9787	1.0000	0.9907	1.0000	0.9718	1.0000	0.9829	1.0000	0.9813	9
P4/Pz/T5	1.0000	0.9816	1.0000	0.9877	1.0000	0.9760	1.0000	0.9829	1.0000	0.9818	3
P4/Pz/T5/O2/ P3	1.0000	0.9839	1.0000	0.9911	1.0000	0.9796	1.0000	0.9862	1.0000	0.9821	5
All Except Fz/F3/C4	1.0000	0.9780	1.0000	0.9926	1.0000	0.9711	1.0000	0.9836	1.0000	0.9830	11
All Except P3	1.0000	0.9813	1.0000	0.9932	1.0000	0.9751	1.0000	0.9857	1.0000	0.9830	13
All Except Fz/F3/C4	1.0000	0.9780	1.0000	0.9926	1.0000	0.9711	1.0000	0.9836	1.0000	0.9830	11
All channels	1.0000	0.9736	1.0000	0.9840	1.0000	0.9673	1.0000	0.9770	1.0000	0.9822	14
All Except P3/F3	1.0000	0.9819	1.0000	0.9926	1.0000	0.9765	1.0000	0.9859	1.0000	0.9837	12
P4/Pz/T5/O2	1.0000	0.9817	1.0000	0.9936	1.0000	0.9756	1.0000	0.9862	1.0000	0.9862	10
P4/Pz/T5/T6/O2/Oz/Cz/F4	1.0000	0.9728	1.0000	0.9892	1.0000	0.9641	1.0000	0.9788	1.0000	0.9767	8

**Figure 7:**
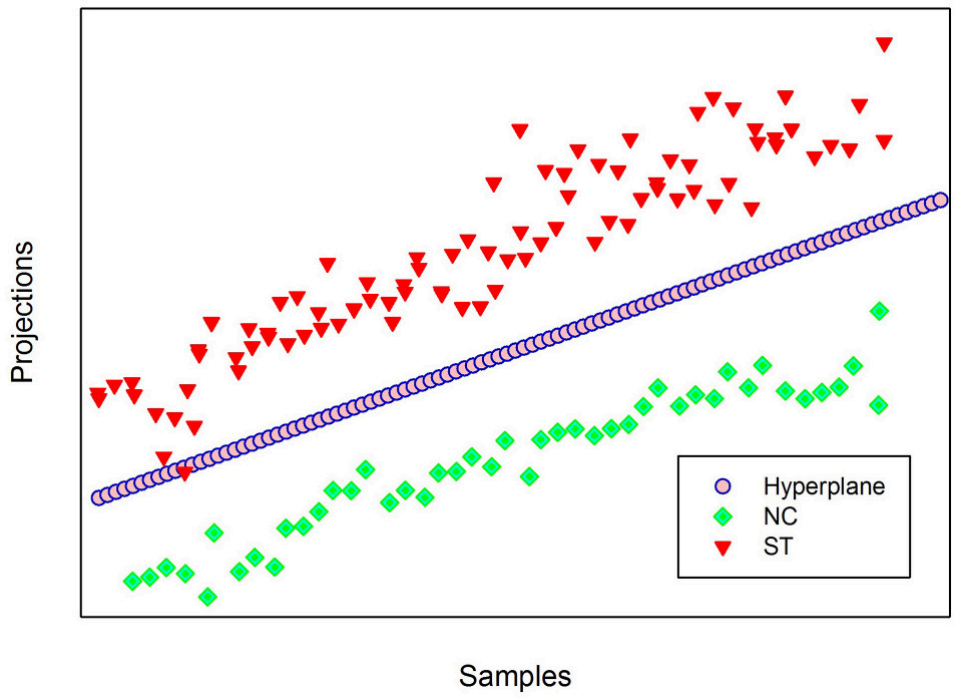
Projection of NC and ST groups on a 2-dimensional coordinate plane.

In linear binary classification, the hyperplane (***W**'* · ***X*** − *b* = 0), is used as a class boundary between the two classes. Where ***W**'* is the transpose of *W* defined in Equation **(**4), ***X*** is the data set to be classified and *b* is a constant. The classifier is assigned a given input sample **X** ϵ *R*^*d*^ (in this case, *d* = 2, 800) according to the sign of (***W**'* · ***X*** − b). If the sign of (***W**'* · ***X*** − b) is positive, ***X*** belongs to the schizotypy group, and if the sign is negative, ***X*** belongs to the control group. Figure 7 visually presents the projection of NC (green) and ST (red) groups on a 2-dimensional coordinate plane. The hyperplane indicated by a broken line creates an ideal boundary between the two groups. For any unseen data, if its projection lies on the lower side of the hyperplane, the new data is classified as NC; and it is classified as ST if its projection lies on the upper side of the hyperplane. The classifier achieved true positive and true negative rates of 98.8 and 100%, respectively.

## Discussion and conclusions

To distinguish individuals with schizotypy from controls, a customized version of the linear discriminant analysis algorithm, called SKLDA, was used. EEG data were fed as input into the discriminant analysis to obtain the discriminant function. The classifier achieved an average of 98.18 % precision, and 98.77% recall rate. Our method achieves a “partially unbiased estimate,” as the data used to develop the discriminant function was not used during validation. Thus, this approach has better generalizability of the results.

The two important tasks that contribute to the enhanced accuracy of our method include estimation of the shrinkage parameter and the LOOCV. Particularly important was the LOOCV procedure, because in this method, the subjects being tested are not involved in the development of the classification functions. This crucial step gives our approach the ability to generalize results for unseen subjects. In addition to the LOOCV, the use of the shrinkage parameter helped to establish a covariance matrix with optimum discriminate information. The minimum classification error occurred at γ = 0.05. When γ = 0.0, since the misclassification rate got its maximum, this verified that the empirical covariance did not contain enough discriminatory information. It also shows the advantage of using the shrinkage linear discriminant analysis over the traditional linear discriminant analysis.

The other important finding of our experiment is the output of the SFS greedy selection algorithm. We found classification accuracy using three channels (Pz, P4, and T5) that showed similar performance to those using 14 channels in identifying individuals with schizotypy from controls, but with significantly reduced computational and calibration cost. This result suggests the possibility that brain activity in the parieto-temporal region can reflect different emotional processing of multisensory stimuli between the two groups. Previous brain imaging studies, using functional magnetic resonance imaging (Calvert, 2001) and magnetoencephalography (Raij et al., 2000) have found that parieto-temporal regions are part of the brain network involved during the audiovisual integration process. More direct evidence from a positron emission tomography study, using test with emotional audio-visual pairs (face expressions and emotional voices), found that the left lateral temporal cortex was related to processing of multisensory stimuli (Pourtois et al., 2005). ERP studies have also found that a positive ERP deflection (P2), a marker of multisensory processing, was mainly localized in the posterior areas (Balconi and Carrera, 2014).

Our study has several advantages. First, using a paradigm with emotional processing demands, which is believed to be affected by schizotypy, allows specific assessment of brain functioning of schizotypy. Because individuals with schizotypy are considered as non-clinical subjects, EEG measurements, with a high level of imposed demands through multimodal emotional processing, might produce significant differences between two groups. In social contexts, moreover, emotional processing ability using multiple sensory modalities is an essential component. While people predominantly rely on the visual modality to judge the emotional states of others, the auditory modality also provides a great deal of emotional information. The ability to extract emotional salience from both visual and auditory signals, and integrate this information, has important implications for successful communication in social life. Therefore, the advantage of our study is the use of an ecologically valid test for assessing brain function of individuals with schizotypy.

Second, a discriminant function is suggested in this paper as a useful diagnostic validator that can reliably distinguish schizotypy from control. Although, self-report questionnaires have been being widely used for schizophrenia spectrum diagnoses, the suggested method has two advantages. First, whereas self-report questionnaires, such as the SPQ, would be affected by the subject's understanding and introspective ability and suffer from response biases, such as the social desirability bias and acquiescence bias (Paulhus and Vazire, 2007), brain activity measurements might be considered more objective and less vulnerable to these types of bias. Because schizotypy is associated with a vulnerability to schizophrenia, it is important to predict which individuals could later develop schizophrenia. This method may have important clinical implications in discriminating individuals with schizotypy, who have a subtle risk and need intervention in advance, among the general population. Therapeutic intervention is effective in the early phase of the disease, and early detection at the beginning of a schizophrenia spectrum disorder results in a direct therapeutic benefit for the potential patient population.

The limitation of our study inferred directly from the use of cross-validation with data of the same session for analysis. Some previous research reported that performance degradation in classification could be observed because of session-to-session transfer (Marcel and Millán, 2007). However, a recent in-depth study on the stability of EEG features for biometrics concluded that EEG signals contain discriminative information that are stable across time (Maiorana et al., 2016). Although, we have not estimated this type of degradation here, we expected the performance degradation to be minimal since EEG is robust against session-to-session transfer.

In sum, a successful method is proposed in this paper to identifying schizotypy from controls based on neurophysiological outcomes of audiovisual emotion perception and shrinkage linear discriminant analysis. Good accuracy and zero false positive rates are among the advantages of our method. The classification accuracy was significantly high (98.22%), in which subjects were correctly classified with an average 98.18% precision and 98.77% recall rates. This method could be useful in early detection of psychosis prone individuals, such as those suffering from schizotypy, as well as helping to elucidate understanding of the progression from schizotypy to schizophrenia.

## Author contributions

JJ designed the experiment, analyzed the data, and wrote the manuscript. TW analyzed the data and wrote the manuscript. EC performed literature research and data analysis and edited the manuscript. YC performed literature research and data analysis and conducted the experiment. JP provided clinical bases of data analysis. HJK supervised the methodological aspects of this study and managed the overall data analysis. HTK supervised the theoretical works and the practical aspects of this study and managed the overall data collection and analysis. All of the authors contributed to the correction of the final manuscript.

### Conflict of interest statement

The authors declare that the research was conducted in the absence of any commercial or financial relationships that could be construed as a potential conflict of interest.
